# Structural and functional characteristics of soil microbial community in a *Pinus massoniana* forest at different elevations

**DOI:** 10.7717/peerj.13504

**Published:** 2022-07-15

**Authors:** Jian Zhang, Ming Xu, Xiao Zou, Jin Chen

**Affiliations:** 1 The Key Laboratory of Plant Resource Conservation and Germplasm Innovation in Mountainous Region (Ministry of Education), Guizhou University, Guiyang, China; 2 Institute of Fungal Resources, Institute of Edible Fungus, College of Life Sciences, Guizhou University, Guiyang, China

**Keywords:** Altitudinal gradient, Metagenome, *Pinus massoniana*, Community structure and function, Subtropical mountain forest, Soil microbial communities

## Abstract

Shifts in forest soil microbial communities over altitudinal gradients have long been attracting scientific interest. The distribution patterns of different soil microbial communities along altitudinal gradients in subtropical mountain forest ecosystems remain unclear. To better understand the changes in soil microbial communities along an altitude gradient, we used Illumina MiSeq metagenome sequencing technology to survey the soil microbial communities in a *Pinus massoniana* forest at four elevations (Mp1000, Mp1200, Mp1400, Mp1600) and in a tea garden in Guizhou Leigong Mountain in Southwestern China. We observed that the richness of bacteria, fungi, and viruses in the soil microbial community changed in a unimodal pattern with increasing elevation while that of Archaea first increased significantly, then decreased, and finally increased again. Euryarchaeota and Thaumarchaeota were the predominant Archaea, Proteobacteria and Acidobacteria were the predominant bacterial groups, Ascomycota and Basidiomycota were the predominant fungal groups, and Myoviridae, Podoviridae, and Siphoviridae were the predominant virus groups. Amino acid transport and metabolism, energy production and conversion, signal transduction mechanisms, and DNA replication, restructuring and repair were the predominant categories as per NOG function gene-annotation. Carbohydrate metabolism, global and overview map, amino acid metabolism, and energy metabolism were predominant categories in the KEGG pathways. Glycosyl transferase and glycoside hydrolase were predominant categories among carbohydrate enzyme-functional genes. Cluster, redundancy, and co-occurring network analyses showed obvious differences in the composition, structure, and function of different soil microbial communities along the altitudinal gradient studied. Our findings indicate that the different soil microbial communities along the altitudinal gradient have different distribution patterns, which may provide a better understanding of the mechanisms that determine microbial life in a mid-subtropical mountain forest ecosystem.

## Introduction

Natural vertical gradients associated with altitude in mountain ecosystems have attracted great interest from ecologists around the world because rapid changes in climate and biological characteristics within a short geographic range are commonly observed in these habitats (Siles & Margesin, 2016). Altitudinal gradients have been used as “natural experiments” to understand how changes in environmental factors such as temperature, precipitation, humidity, solar radiation, and atmospheric pollution deposits affect changes in the complex biota (plant, animal, microbial) communities in mountain terrains (Körner, 2007). The mechanism underlying changes in species richness along an altitudinal gradient has always been a controversial issue in ecological and biogeographic studies. Although there are reports of diminishing patterns or single-peak patterns for animal and plant mechanisms responsive to elevation, such differences may show stronger variation for microorganisms among different regions, including no trend, a decline, a single peak, or a concave or other distribution patterns associated with changes in altitude (Liu et al., 2018; Siles & Margesin, 2016). In recent years, research on altitudinal patterns of microbial (*i.e*., Bacteria, Fungi, ectomycorrhizal fungi, *etc*.) diversity has gradually increased by using high-throughput sequencing technology (Geml et al., 2017; Jarvis, Woodward & Taylor, 2015; Liu et al., 2018; Schoen, Nieselt & Garnica, 2018; Shen et al., 2020; Wang et al., 2015; Zhao et al., 2022; Zhou et al., 2021). Soil microorganisms are important biological components associated with aboveground interactions of terrestrial ecosystems (Bahram et al., 2018), recognized as key drivers of litter decomposition, plant growth, soil nutrient cycling, and biogeochemical processes (Bardgett & van der Putten, 2014; Schloter et al., 2018; Thakur & Geisen, 2019; van der Heijden & Wagg, 2013). Due to the importance of forest soils as both sinks and potential sources of carbon, as well as to their predicted sensitivity to climate change, microbial ecologists have been struggling to indicate what is the interaction mechanism between environment factors (topography, climate, vegetations and soil) and soil microbial communities (diversity and function) in forest ecosystems (Lladó, López-Mondéjar & Baldrian, 2018). Topography, as a long-term and constant factor, has a major impact on ecosystem dynamics, and changes in topography may lead to changes in soil microbial communities (Qiu et al., 2012; Tajik, Ayoubi & Lorenz, 2020; Zhang et al., 2013). From a biological perspective, topographic parameters can provide accurate and valuable information for predicting the distribution of the biodiversity of soil microbial communities (Lladó, López-Mondéjar & Baldrian, 2018; Tajik, Ayoubi & Lorenz, 2020). Therefore, promoting the study of the mechanism underlying the distribution of soil microorganisms along altitudinal gradients in mountain ecosystems should prove useful for scientifically-based management of mountain ecosystems (Geml et al., 2017; Jarvis, Woodward & Taylor, 2015; Körner, 2007; Liu et al., 2018; Schoen, Nieselt & Garnica, 2018; Siles & Margesin, 2016; Wang et al., 2015).

Mountain terrains are very common in Southwest China, which increase habitat heterogeneity while providing an important foundation for biodiversity richness (Myers et al., 2000). *Pinus massoniana* (Masson pine) is an important native tree species and the most widely distributed pine tree in Southern China, with strong adaptability and tolerance to harsh environments (Lei et al., 2018). In fact, *P. massoniana* forest is the main vegetation type in Southern China with key ecological and economic importance, accounting for 6.1% and 4.0% of the total area and volume of tree forests in China, respectively (Huang et al., 2020). Masson pine forests are distributed along elevation gradients and thus provide natural experimental locations for studying the elevation-associated mechanism underlying the distribution of soil microbial communities.

In the present study, we aimed (i) to describe the taxonomic composition of different microbial communities (Archaea, Bacteria, Fungi, and Viruses) that those soils harbor (using Illumina MiSeq metagenome sequencing) along an altitudinal gradient; (ii) to analyze the diversity and functional structure of soil microbial communities at different altitudes; (iii) to assess the relationship between the diversity and function of soil microbial communities in a typical mid-subtropical mountain (Leigong Mountain) Masson pine forest ecosystem in southwestern China (Guizhou Province). This study will make us better understand the structural and functional characteristics of soil microbial community in subtropical mountain forests.

## Materials and Methods

### Site description

The Leigong Mountain Nature Reserve (26°15′–26°32′ N; 108°5′–108°24′ E) was first established as a Provincial Nature Reserve in 1982 and then promoted to a National Nature Reserve in 2001. Leigong Mountain with 83% forest coverage belong to the typical mid-subtropical mountain forest ecosystem (Zhang et al., 2020). Leigong Mountain belongs to the humid climatic region of the mid-subtropical monsoon mountainous region. The annual rainfall is 1,300∼1,600 mm; the annual average temperature is about 9.2 °C and 16.3 °C at the top and at the foot of the mountain, respectively, with an annual average temperature drop rate of 0.5 °C per 100 m. The soils are mainly acidic mountain yellow soils and mountain yellow brown soils, with a deep soil profile. The zonal vegetation belongs to the humid evergreen broad-leaved forest in the eastern mid-subtropical zone, where it is distributed below 1,350 m a.s.l., while the mountain evergreen deciduous broad-leaved mixed forest is distributed between 1,350 and 2,100 m a.s.l., above which the alpine shrub is observed. Leishan county also is one of the main tea producing areas in Guizhou province (the organic tea area is 653.2 hm^2^, and the annual tea products is 3,950 t), where tea garden are typical artificial shrub vegetation in mountain ecosystem.

### Study site and soil sampling

**Figure 1:**
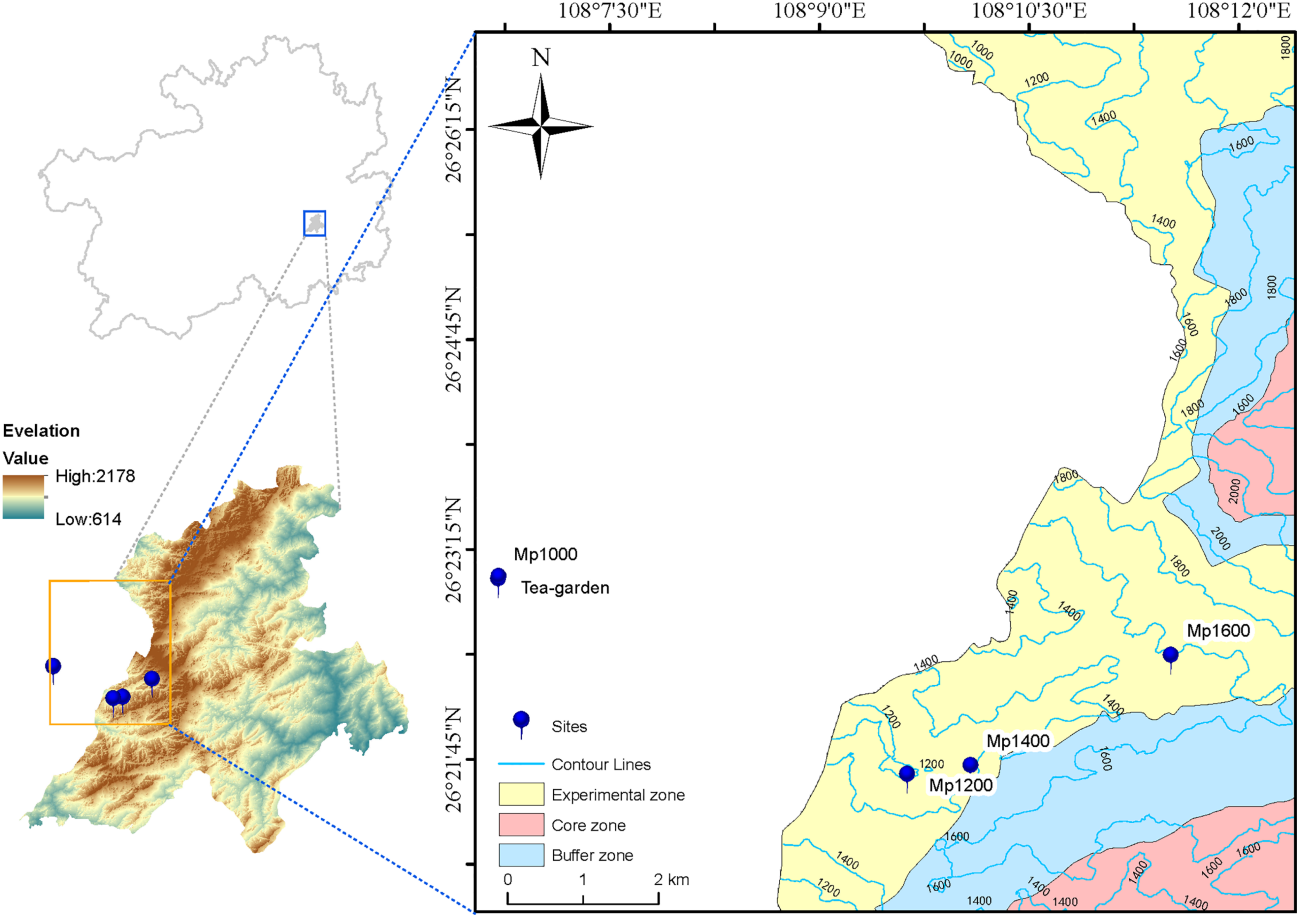
Map of study area and sampling sites.

In June 2019, four sites were selected along the altitudinal gradient established on the *P. massoniana* forest in the Leigong Mountain at 1,000, 1,200, 1,400, and 1,600 m a.s.l. and named Mp1000, Mp1200, Mp1400, and Mp1600, respectively. An artificial tea garden (Tea-garden) was selected as the control plot at 1,000 m a.s.l. (Fig. 1). One vegetation survey plot with 20 m × 20 m in size was also set at each altitude. Information on site conditions (Table 1), plant communities, and litter was obtained for each plot. Soil samples were excavated from the 0–20 cm topsoil layer at six points located along an “S” path drawn within each plot, and thoroughly mixed into one composite sample. Five soil samples were collected (one from each survey plot plus one from the control plot). For each sample, 500 g of soil was sieved though a 2-mm mesh and air-dried for physicochemical analyses; additionally, 50 g of soil was encapsulated in a plastic tube and kept at −80 °C for microbial DNA extraction.

**Table 1:** Location and dominant species of study sites.

Sites	Latitude and longitude	Altitude (m)	Slope (°)	Aspect (°)	Dominant species
Mp1000	108°06′42.08″, 26°23′00.72″	1,008	12	121	*Pinus massoniana + Cunninghamia Lanceolata*
Mp1200	108°10′02.83″, 26°21′29.72″ 108°09′37.60″, 26°21′36.92″	1,198	10	160	*P. massoniana*
Mp1400	108°10′04.86″, 26°21′40.68″	1,394	15	141	*P. massoniana*
Mp1600	108°11′30.85″, 26°22′27.92″	1,596	7	165	*P. massoniana + C. Lanceolata*
Tea-garden	108°06′42.21″, 26°23′01.73″	991	3	120	*Camellia sinensis*

### Soil analysis and microbial DNA extraction

Soil pH was determined at a ratio of 1:2.5 (soil to water, w/w). Soil organic carbon (SOC) was determined using the K_2_Cr_2_O_7_ titration method (Walkley–Black method). Total nitrogen (TN) was measured using the semi-micro Kjeldahl method. Total phosphorus (TP) was determined colorimetrically after wet digestion with H_2_SO_4_ + HClO_4_ (Xu et al., 2014).

### Microbial DNA extraction and metagenome sequencing

Total genomic DNA was isolated from 0.50 g of soil using the MoBio Ultraclean Soil DNA Isolation Kit (MoBio Laboratories, Carlsbad, CA, USA) following the instructions of the manufacturer. Extracted DNA was stored at −80 °C until use. Tenfold diluted DNA samples were then used for metagenome analysis on the Illumina MiSeq platform (Illumina Inc., San Diego, CA, USA) according to the standard protocols of Majorbio Bio-Pharm Technology Co. Ltd. (Shanghai, China).

The MiSeq platform was used to perform parallel mixing and sequencing of DNA multiple samples. The sequence of each sample was introduced with an index tag sequence indicating sample source information. Software “Seqprep” was used to cut the 3′ end and 5′ adapter sequence at the end. Software “Sickle” was used to remove sequences less than 50 bp; the average quality value was below a certain threshold (default 20) and N-base reads; high-quality pair-end reads and single-end reads were retained (Pabinger et al., 2014; Schirmer et al., 2015). We then used “fastp” (https://github.com/OpenGene/fastp) software to perform quality control processing on the original sequencing data to obtain high-quality control data (clean data). Software “Megahit” (https://github.com/voutcn/megahit) was used to assemble sequences with different sequencing depths (Li et al., 2015) and to preserve sequences with assembly contigs >300 bp. MetaGene (http://metagene.cb.ku-tokyo.ac.jp/) software was then used to predict open reading frames (ORF) of contigs ≥100 bp (gene sequences) in clean sequences (Noguchi, Park & Takagi, 2006). Using CD-HIT (http://www.bioinformatics.org/cd-hit/) software, clustering (identity ≥90% and coverage ≥90%) was performed on the predicted gene sequences of all samples, taking the longest gene in each class as a representative sequence to construct a non-redundant gene set (Fu et al., 2012). SOAPaligner software was used to compare the high-quality reads of each sample with the non-redundant gene set (identity ≥95%) and to obtain gene abundance information in the corresponding samples (Luo et al., 2012). For species identification, BLASTp (http://blast.ncbi.nlm.nih.gov/Blast.cgi) software was first used to blast the obtained gene sequences against the non-redundant (NR) protein sequence database (Altschul et al., 1997) and the evolutionary genealogy of genes: Non-supervised Orthologous Groups (eggNOG) database (http://eggnog.embl.de/) with the expected e-value for the functional annotation set to 1e−5 (Jensen et al., 2008). The Kyoto Encyclopedia of Genes and Genomes (KEGG) Database and an e-value of 1e−5 were used for functional annotation (Du et al., 2014). The Carbohydrate-active enzymes (CAZy) database (http://www.cazy.org/, Version 5.0) and an e-value of 1e−5 were used for active carbohydrate enzymes functional annotation (Lombard et al., 2014). Fungal guilds were assigned using the FUNGuild platform (http://www.funguild.org/) (Nguyen et al., 2016).

The number of total and unique species and the functional genes of Archaea, Bacteria, Fungi, and Virus groups in the different soil samples were determined and, using the VennDiagram R package (https://www.r-project.org) a Venn diagram was produced for the numbers of Archaea, Bacteria, Fungi, and Viruses in the soil microbial community. A horizontal community histogram, also produced in R using cluster analysis, was based on the similarity of soil microbial community between samples. Similarity results of the soil microbial community among different soil samples are presented as a heatmap.

The correlation between the soil microbial community matrix and the function matrix among the different samples was also tested. The soil microbial community species-level matrix based on the Bray-Curtis distance algorithm was used for redundancy analysis (RDA). The “networks” software was used for co-occurrence network analysis (Barberan et al., 2012). Soil environmental factors were analysed using One-way analysis of variance (ANOVA) in SPSS 22.5 (https://www.ibm.com/analytics/spss-statistics-software). Finally, Sigma Plot 12.5 and Adobe Illustrator 2020 (https://www.adobe.com/products/illustrator.html) software were used for figure drawing.

## Results

### Composition and structure of soil microbial communities

A total of 452,526,750 original sequences were obtained from the metagenomic sequencing of soil microorganisms in the *P. massoniana* forest along the altitudinal gradient established. After quality control, 447,074,316 high-quality sequences were generated and then divided into different soil microbial groups, including Archaea, Bacteria, Fungi, and Viruses. There were 62,208,980 high-quality sequences and each sample produced 9,960,236~16,161,802 high-quality sequences. After metagenomic sequencing, BALSTp on the NR database allowed identifying 13,307 species of soil microorganisms. These included 912 Archaea, 11,576 Bacteria, 417 Fungi, and 402 Viruses (Table 2).

**Table 2:** The number of metagenomic sequencing by NR species annotation.

Microbe	Phylum	Class	Order	Family	Genus	Species
Archaea	12	24	38	60	144	912
Bacteria	82	135	251	466	1,864	11,576
Fungi	10	33	74	154	254	417
Viruses	1	1	4	17	60	402
Total	105	193	367	697	2,322	13,307

The changes in richness and in Shannon’s index of soil microbial groups at the different altitudes selected at the study site showed different trends (Table 3). In terms of species richness, the abundances of soil Bacteria, Fungi, and Viruses exhibited a “unimodal” pattern with altitude, while Archaea showed a major peak at Mp1200 (752) and a secondary peak at Mp1600 (716). With increasing altitude, Shannon’s index values increased for Archaea and Fungi but decreased for Bacteria; for Viruses, no obvious pattern was found.

**Table 3:** Alpha diversity of soil microbial community.

Sites	Richness				Shannon index			
	Archaea	Bacteria	Fungi	Viruses	Archaea	Bacteria	Fungi	Viruses
Mp1000	689	9,993	292	125	7.29	9.08	7.3	4.72
Mp1200	752	10,172	297	166	7.64	8.62	7.16	4.45
Mp1400	698	10,247	318	138	7.85	8.97	7.29	4.91
Mp1600	716	10,114	297	118	7.86	8.9	7.12	4.06
Tea-garden	697	10,348	354	225	7.03	8.45	7.51	5.7

**Figure 2:**
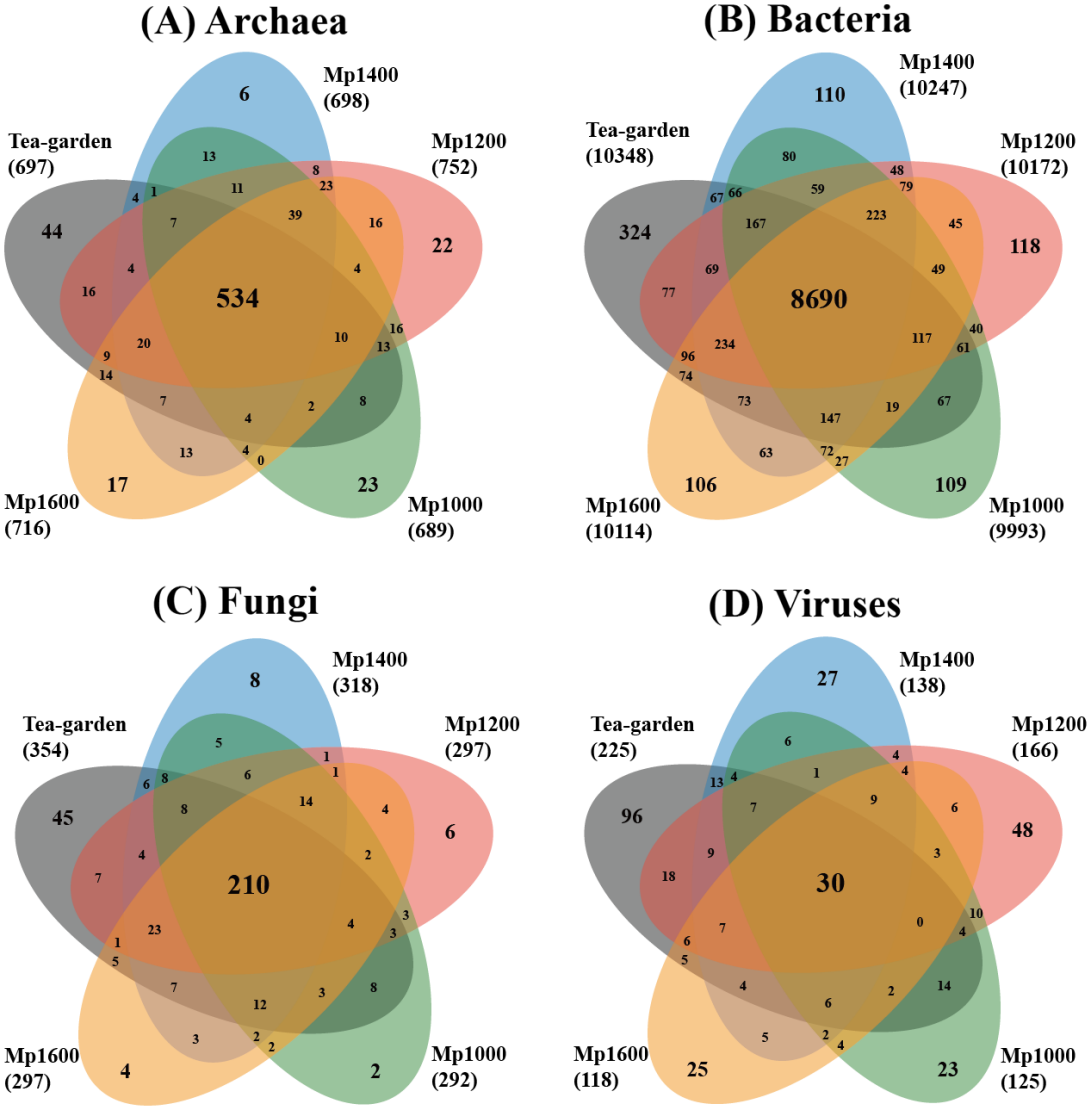
Venn diagram of exclusive, shared species, and total species associated with (A) archaea, (B) bacteria, (C) fungi and (D) viruses in soil microbial communities at different elevations in a *Pinus massoniana* forest.

The distributions of exclusive and common species of the different soil microbial groups showed obviously different profiles (Fig. 2). Common species of Archaea, Bacteria, Fungi, and Viruses accounted for 58.6%, 75.1%, 50.4%, and 7.5% of the soil microbial groups, respectively, whereas exclusive species of Archaea, Bacteria, Fungi, and Viruses accounted for 12.3%, 6.6%, 15.6%, and 54.5%, respectively. The spatial distribution patterns of the different soil microbial groups also differed across the altitudinal gradient. The ratio of exclusive and common species for Archaea and Fungi were similar, but with obvious differences of the ratio for Bacteria or Viruses.

**Figure 3:**
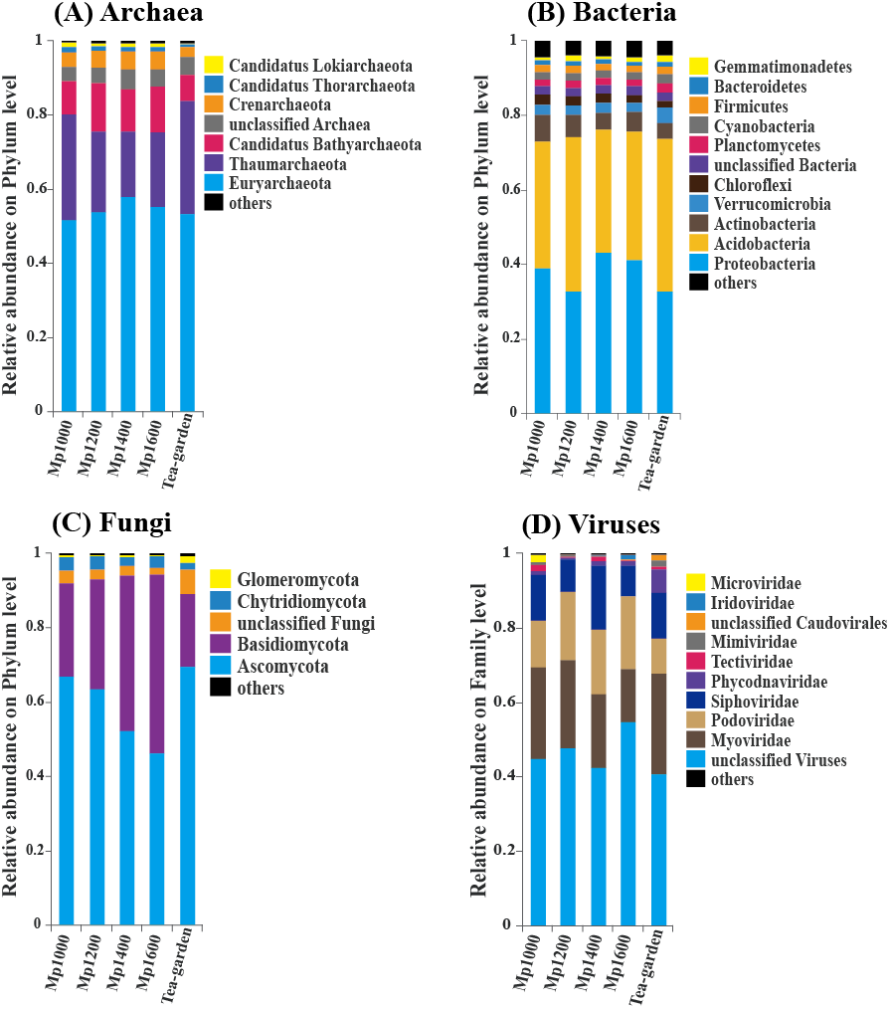
The relative abundance of (A) Archaea, (B) Bacteria, (C) Fungi, (D) Viruses in soil microbial communities across an altitudinal gradient in a *Pinus massoniana* forest.

Euryarchaeota and Thaumarchaeota were the predominant groups of soil Archaea in the *P. massoniana* forest surveyed across the selected altitudinal gradient. The order of relative abundance of these two groups at the five sites was: Tea-garden (84.1%) > Mp1000 (80.3%) > Mp1200 (75.8%) > Mp1400 (75.6%) > Mp1600 (75.5%), thus showing a decreasing trend from 1,000 to 1,200 m altitude. Euryarchaeota and Thaumarchaeota were followed in relative abundance by Candidatus Bathyarchaeota, Crenarchaeota, Candidatus Thorarchaeota, Candidatus Lokiarchaeota, Candidatus Micrarchaeota, Candidatus Korarchaeota, Candidatus Parvarchaeota, Candidatus Nanohaloarchaeota, and Nanoarchaeota (Fig. 3A).

Proteobacteria and Acidobacteria were the predominant groups of Bacteria. The order of relative abundance of these two bacterial groups at the five sites was: Mp1000 (73.1%) < Tea-garden (74.0%) < Mp1200 (74.3%) < Mp1600 (76.0%) < Mp1400 (76.2%), thus showing an increasing trend with increasing altitude. The bacterial groups following in relative abundance were Actinobacteria, Verrucomicrobia, Chloroflexi, Planctomycetes, Cyanobacteria, Firmicutes, Bacteroidetes, Gemmatimonadetes, and other bacteria (Fig. 3B).

Ascomycota and Basidiomycota were the predominant groups of Fungi. The order of relative abundance of these two fungal groups at the five sites was: Tea-garden (89.4%) < Mp1000 (92.2%) < Mp1200 (93.3%) < Mp1400 (94.2%) < Mp1600 (94.4%), thus showing an increasing trend with increasing altitude. Moreover, ascomycetes increased with elevation and their relative abundance decreased significantly whereas basidiomycetes showed an obvious increase with elevation. The relative abundances of the two groups showed increasing trends with increasing altitude. The fungal groups following in relative abundance were Chytridiomycota, Glomeromycota, other Fungi, and unclassified Fungi (Fig. 3C).

Myoviridae, Podoviridae, and Siphoviridae were the predominant virus groups. The order of relative abundance of these groups at the five sites was: Mp1600 (42.1%) < Tea-garden (48.7%) < Mp1000 (49.6%) < Mp1200 (50.8%) < Mp1400 (54.5%). The groups following in relative abundance were Phycodnaviridae, Tectiviridae, Mimiviridae, unclassified virus, Caudovirales, and other viruses (Fig. 3D).

**Figure 4:**
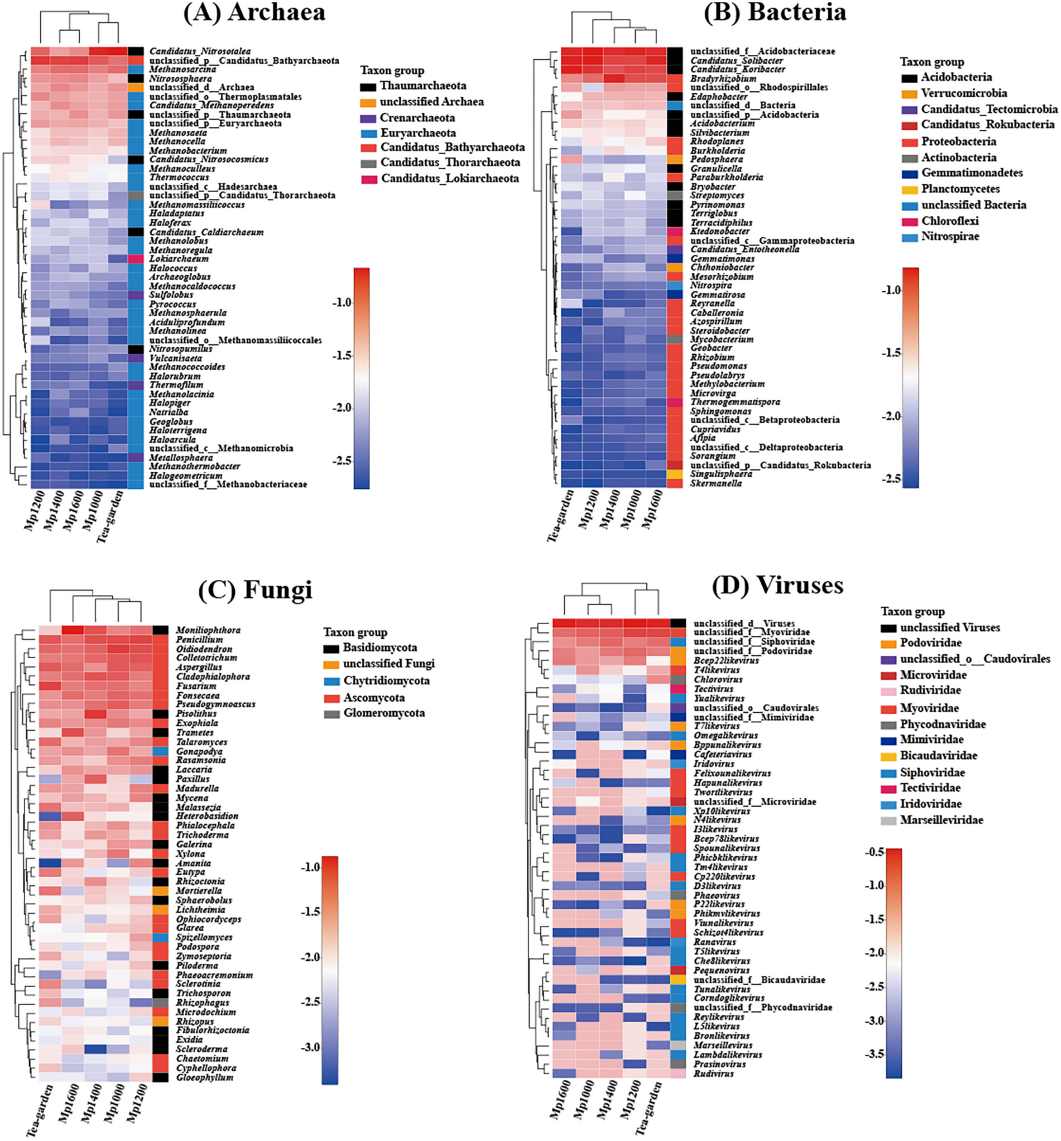
Relationship between (A) Archaea, (B) Bacteria, (C) Fungi, (D) Viruses genera present across an altitudinal gradient in a *Pinus massoniana* forest.

Clustering of the different soil microbial groups (Archaea, Bacteria, Fungi, and Viruses) at the genus level showed significant differences with altitude. Overall, *Candidatus_Nitrosotalea*, *unclassified_p_Candidatus_Bathyarchaeota* (from phylum to genus), *Methanosarcina*, *Nitrososphaera*, and *unclassified_d_Archaea* (from domain to genus) were the predominant archaeal genera (Fig. 4A), while *unclassified_f_Acidobacteriaceae* (from family to genus), *Candidatus_Solibacter*, *Candidatus_Koribacter*, and *Bradyrhizobium* were the predominant bacterial genera (Fig. 4B). *Moniliophthora*, *Penicillium*, *Oidiodendron*, *Colletotrichum*, *Aspergillus*, and *Cladophialophora* were the predominant fungal genera (Fig. 4C). *Unclassified_d_Viruses* (from domain to genus), *unclassified_f_Myoviridae* (from family to genus), *unclassified_f_Siphoviridae* (from family to genus), and *unclassified_f_Podoviridae* (from family to genus) were the predominant virus genera (Fig. 4D).

### Functional gene composition and structure of soil microbial communities

**Figure 5:**
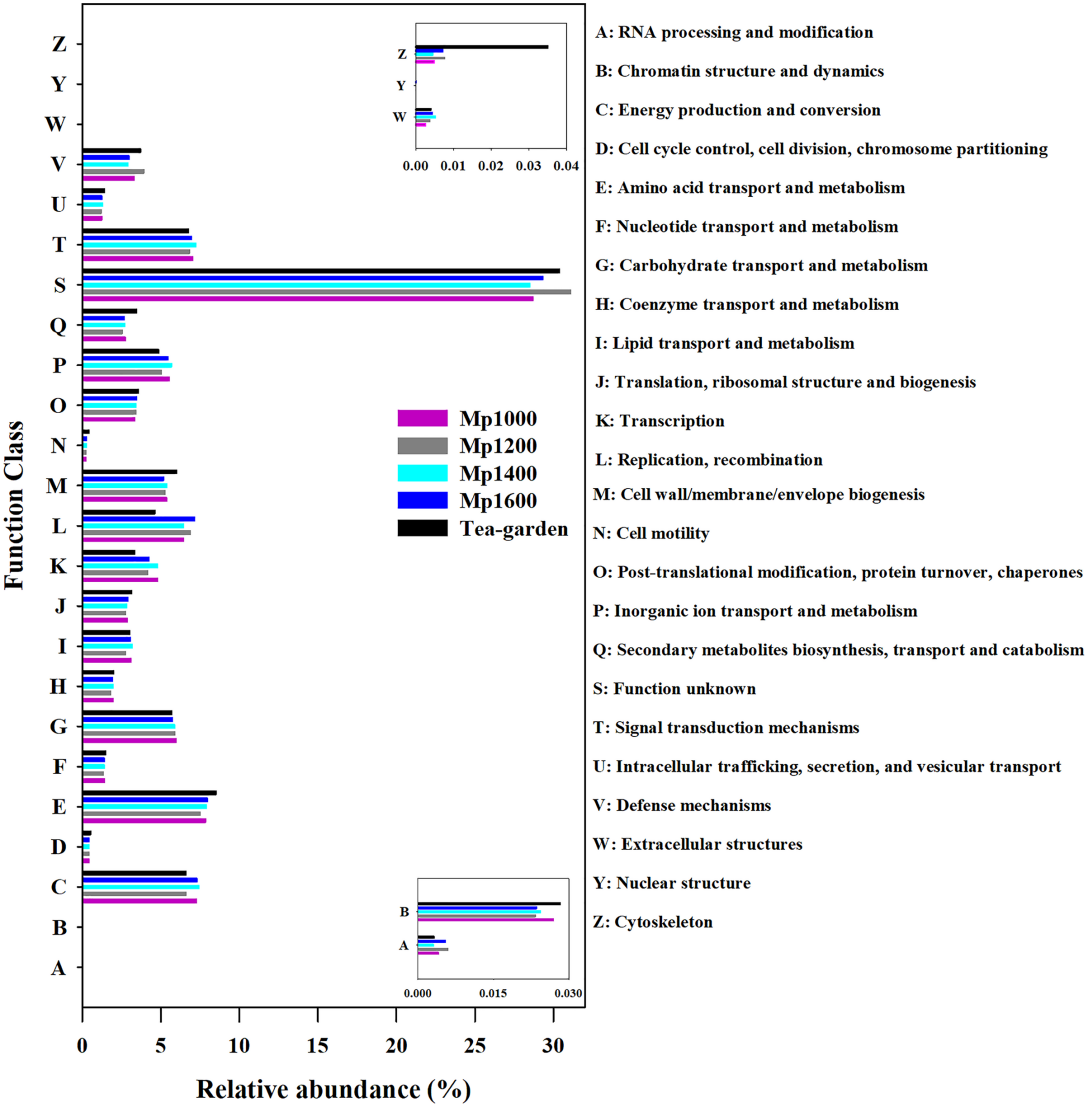
NOG functional gene composition of soil microbial communities at different elevations in a *Pinus massoniana* forest.

Based on the eggNOG database, the soil microbial community functional annotation was determined for the *P. massoniana* forest at different altitudes (Fig. 5). The main groups of functional genes were related to amino acid transport and metabolism (7.51–7.98%), energy production and transformation (6.62–7.46%), signal transduction mechanisms (6.76–7.24%), and DNA replication, recombination, and repair (6.46–7.16%). These dominant functional gene categories are important for energy production and cell metabolism. Genes with unknown functions accounted for 30% (28.58–31.16%) with the most abundant of all functional genes classified.

**Figure 6:**
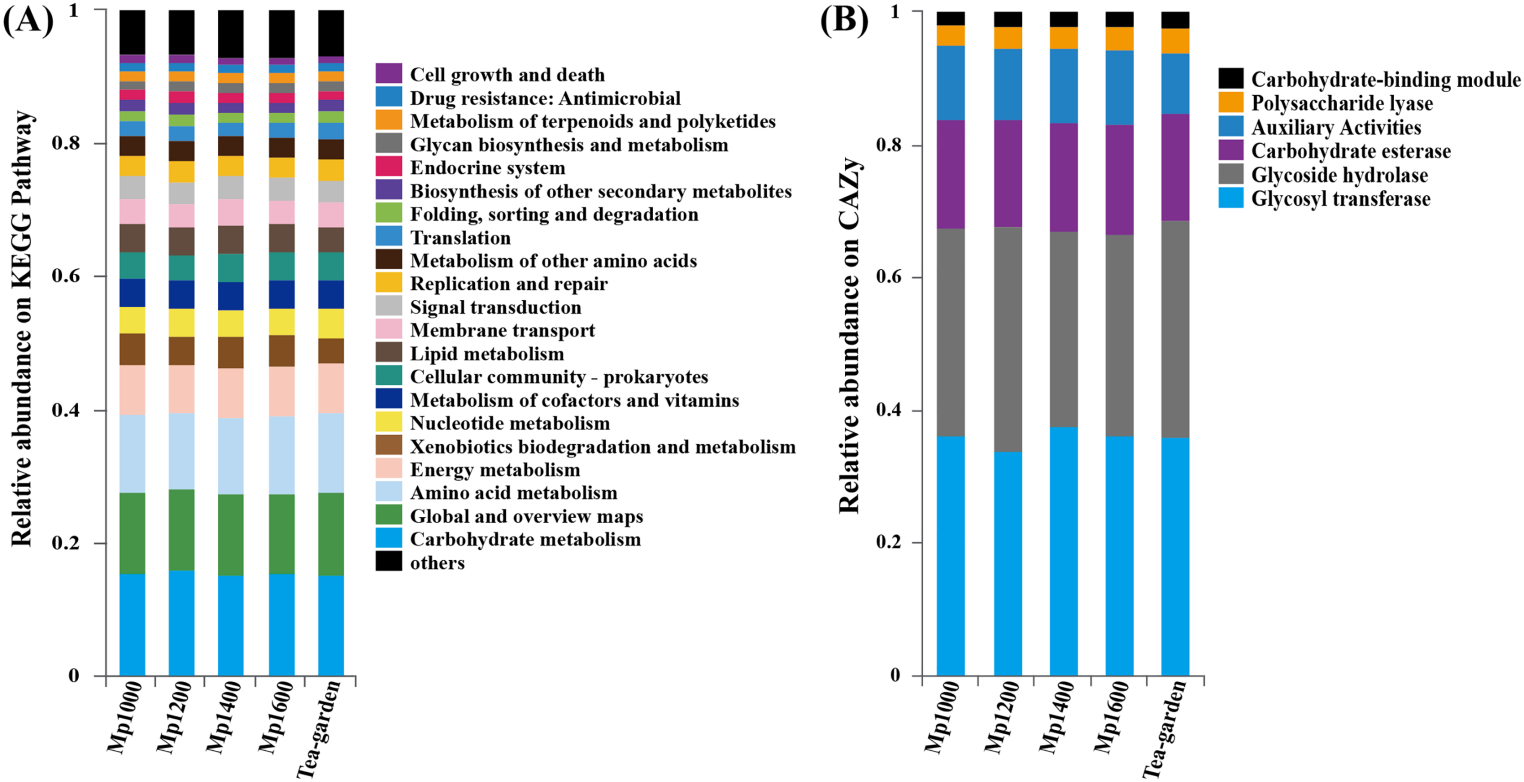
KEGG pathway (A) and CAZy (B) functional gene classification for soil microbial communities at different elevations in a *Pinus massoniana* forest.

A high representation of carbohydrate metabolism (12.22–13.00%), global and overview map (11.51–11.62%), amino acid metabolism (10.23–10.67%), and energy metabolism (7.51–7.80%) was observed when the KEGG database was applied to the functional annotation into putative pathways (Fig. 6A). Glycosyl transferase (GT, 34.59–38.58%) and glycoside hydrolase (GH, 28.98–33.59%) were most represented carbohydrate active enzymes (CAZy), followed by carbohydrate esterase (CE, 15.91–16.28%), coenzyme activity (AA, 10.43–10.92%), and polysaccharide lyase (PL, 3.00–3.64%), combined with the carbohydrate Module (CBM, 1.98–2.14%) (Fig. 6B).

**Figure 7:**
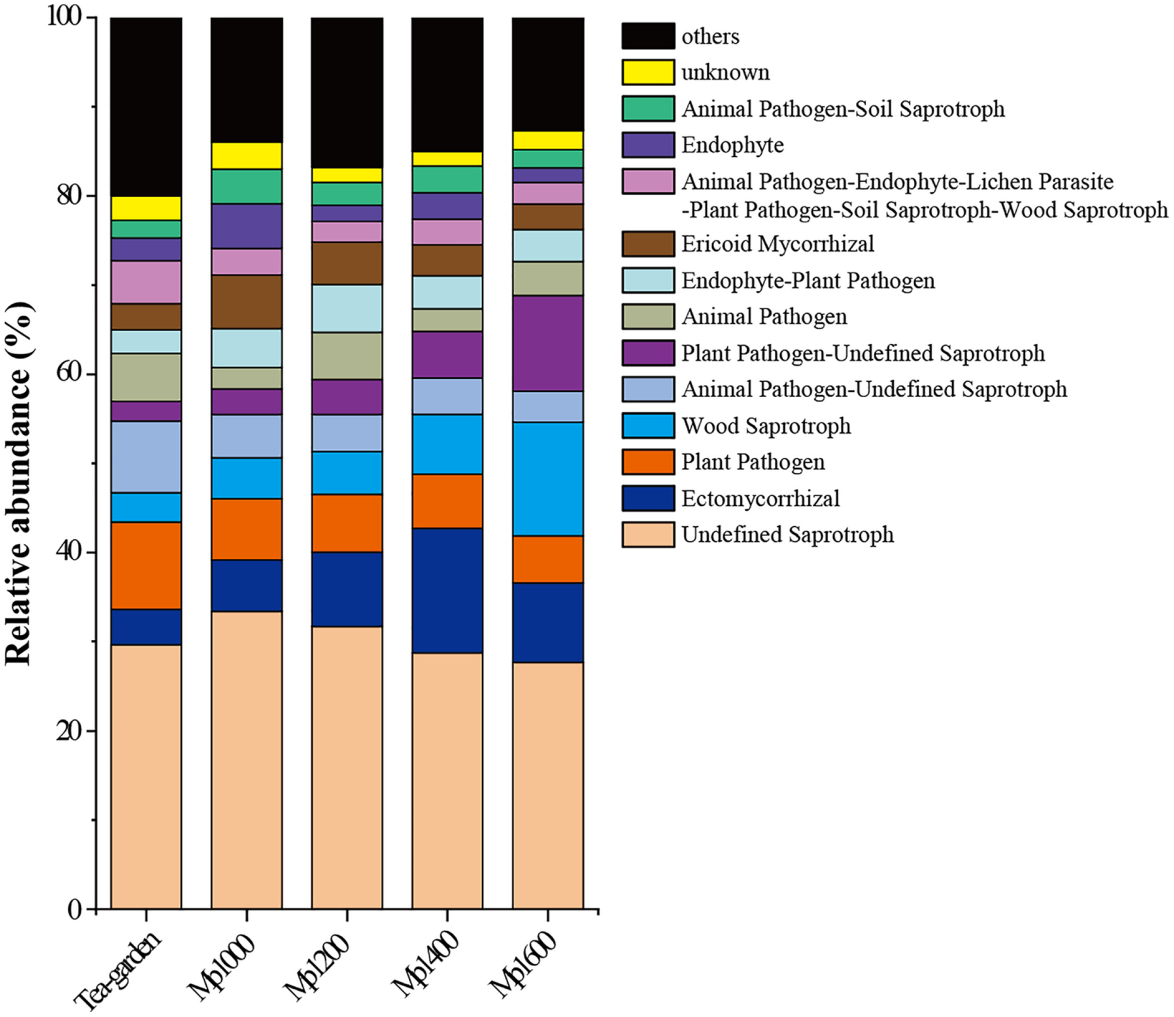
FUNGuild function prediction for soil fungal communities at different elevations in a *Pinus massoniana* forest.

Saprophytic and parasitic trophic mode made up the two largest proportions in soil fungal communities in the *P. massoniana* forest; while Ectomycorrhizal fungi and Ericoid mycorrhizal fungi were dominant groups in the symbiotic trophic mode. The relative abundances of different functional groups in soil fungal communities in the *P. massoniana* forest showed varying trends with increasing altitudes (Fig. 7), such as undefined saprotroph, plant pathogen, animal pathogen-undefined saprotroph, and ericoid mycorrhizal fungi showed a decreasing trend with increasing altitudes, while wood saprotroph, plant pathogen-undefined saprotroph, and Ectomycorrhizal fungi showed an increasing trend with increasing altitudes (Fig. 7). These results suggest that specific functional group in soil fungal communities may be better indicators of forests in terms of elevation changes.

### Similarity of species and functions among soil microbial communities

**Figure 8:**
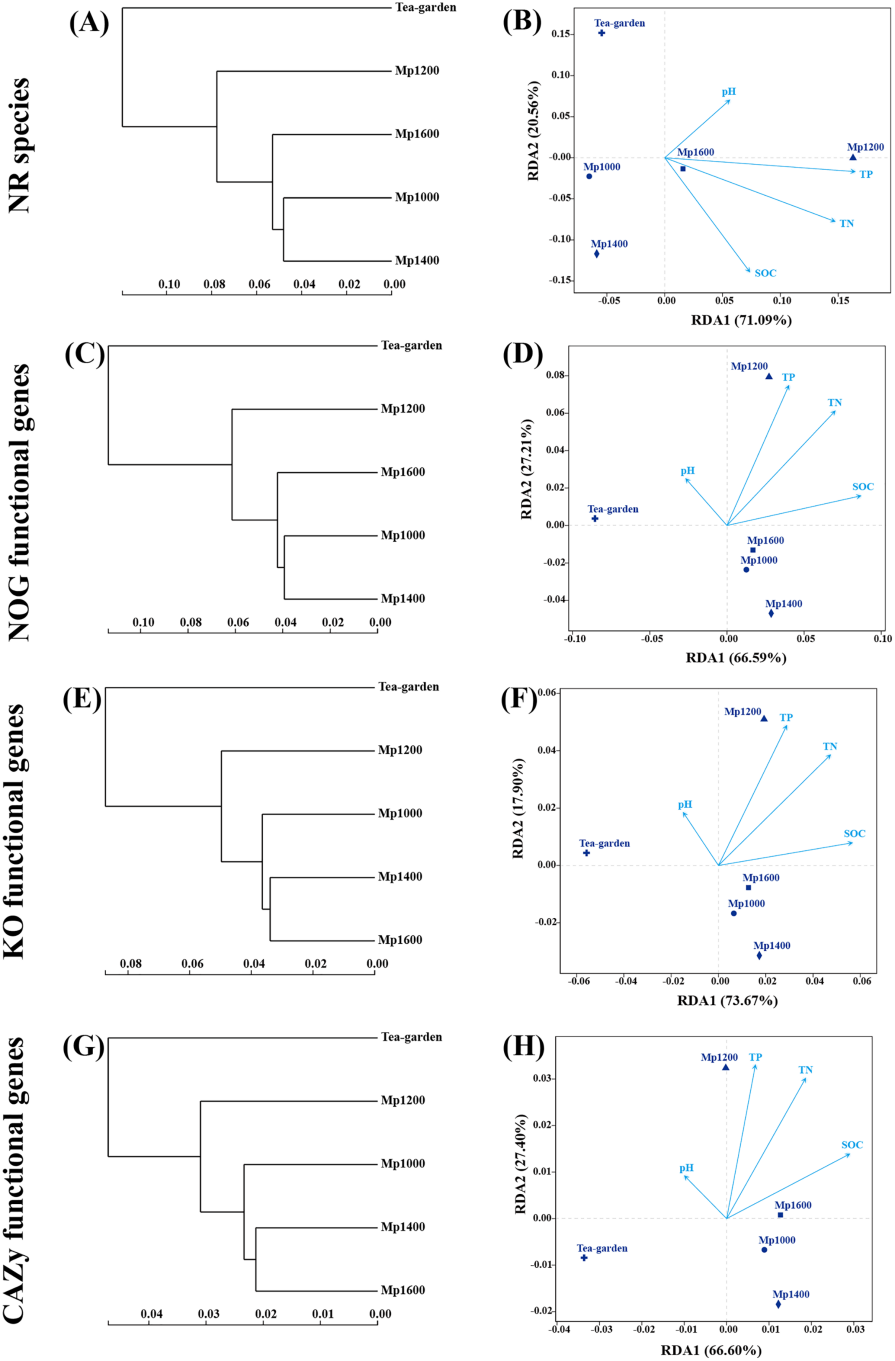
NR species, NOG, KO, and CAZy classification of functional genes of soil microbial communities by clustering tree (A, C, E, G) and RDA analyses (B, D, F, H) at different elevations in a *Pinus massoniana* forest.

The similarity and dissimilarity of species as per NR, NOG, KO, and CAZy functional genes among all sample sites were compared to results obtained from similarity clustering analysis and RDA (Fig. 8). The similarity of species and functions of soil microbial communities along the altitudinal gradient in the *P. massoniana* forest was as described above, *i.e*., the RDA ordination of soil microbial communities at different altitudes based on functional gene composition showed a good level of consistency with the ordination based on species identification. The distribution of identified species, NOG, KO, and CAZy functional genes of soil microbial community at MP1000, MP1400, and MP1600 were clearly separated from those observed at MP1200 (Fig. 8B). Furthermore, a significant difference was observed between the *P. massoniana* forest and the tea garden surveyed.

### Co-occurrence patterns of the different soil microbial groups

**Figure 9:**
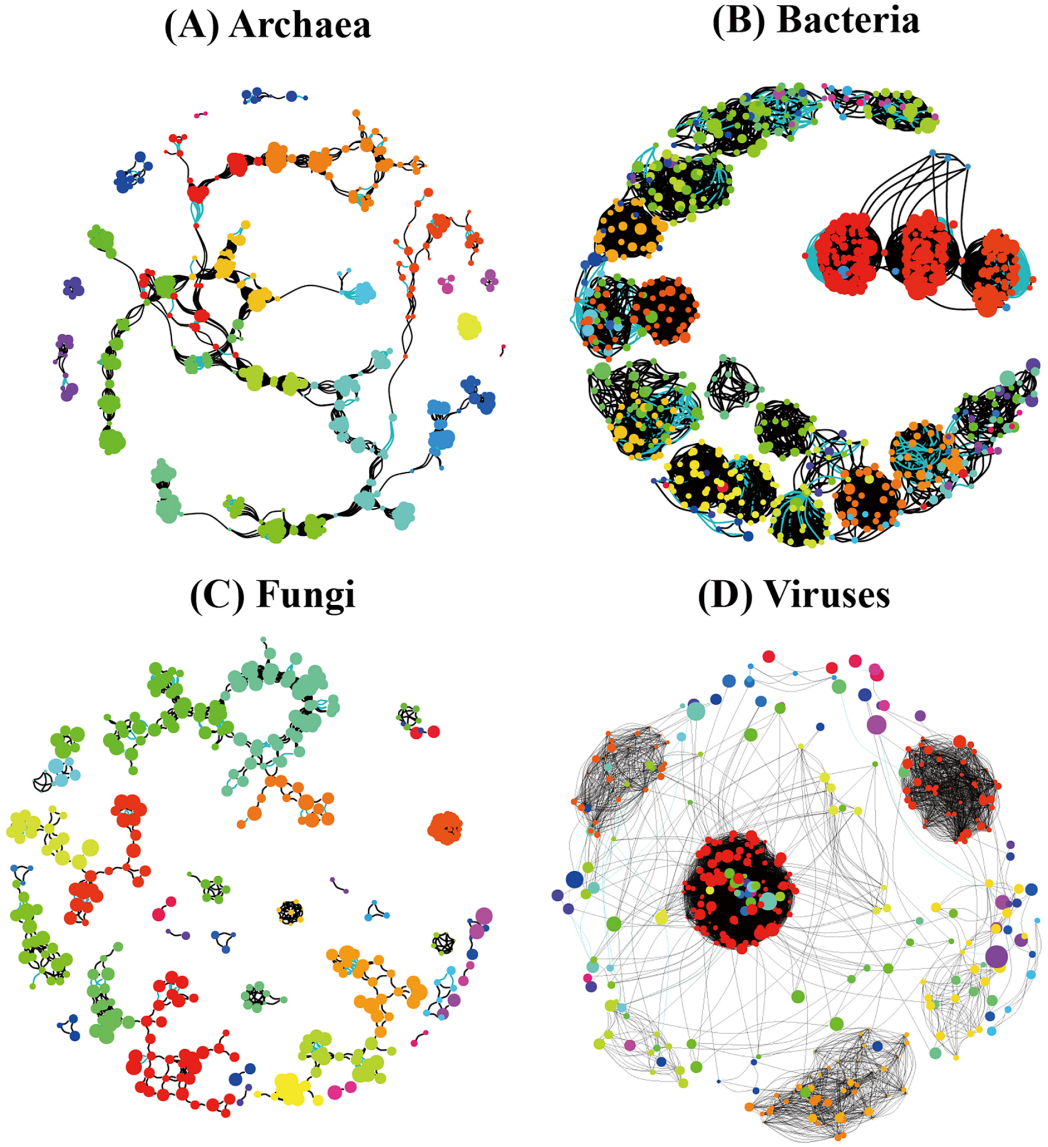
Species co-occurrence networks of non redundant species of the different soil microorganisms ((A) Archaea; (B) Bacteria; (C) Fungi; (D) Viruses) in *Pinus massoniana* forest.

In order to understand the correlation between species of the different soil microbial communities, we detected significant differences among different soil microbial groups (Archaea, Bacteria, Fungi, and Viruses) and their corresponding random networks (Fig. 9) in terms of average degree, modularity, number of communities, and degree centrality were found (Table 4). Modularity ranked as follows: Archaea (0.866) > Bacteria (0.794) > Fungi (0.698) > Viruses (0.539), while degree centrality showed the following ranking: Bacteria (0.181) > Viruses (0.155) > Fungi (0.087) > Archaea (0.048) (Table 4). Overall, the co-occurrence networks of archaeal and fungal species showed a relatively high similarity, while the co-occurrence network of bacterial and viral species showed a relatively high similarity.

**Table 4:** Topological properties of soil microbiome co-occurrence network.

Network properties	Archaea	Bacteria	Fungi	Viruses
Number of nodes	527	748	399	236
Positive edges	2,589	12,308	1,762	6,952
Negative edges	494	663	144	15
Average degree	11.7	34.7	9.6	37.1
Modularity	0.866	0.794	0.698	0.539
Number of communities	23	53	38	34
Degree centralization	0.048	0.181	0.087	0.155

## Discussion

The relationship between altitudinal distribution patterns and maintenance of biodiversity is currently a highly controversial topic in the field of biodiversity and ecosystem functions. The Leigong Mountain belongs to a typical mid-subtropical mountain forest ecosystem in southwestern China, and it is a National Nature Reserve (Zhang et al., 2020) with a well-preserved ecological environment, thus providing an ideal site for “the *in-situ* experiment” reported herein. In addition, previous studies at this mountain ecosystem provided important information on soil properties and vegetation types along the elevation gradient of Leigong Mountain, indicating that the different soil and vegetation types have obvious spatial boundaries (Chen et al., 2012; Zhang, Qiu & Mao, 2019). Therefore, we hypothesized that the different soil microbial communities (Archaea, Bacteria, Fungi, and Viruses) could have different distribution patterns along the different elevations of this typical mid-subtropical mountain forest ecosystem.

*P. massoniana* is a native and widely distributed tree species that is well adapted to the regional climatic conditions (Lei et al., 2018); this ensured the selection of representative *P. massoniana* forest plots along the altitudinal gradient established for conducting the survey reported herein. Soil microbial communities can be diagnosed by metagenome technology, which can annotate microorganisms to the species level, and detect Archaea, Bacteria, Fungi, and Viruses in soil microbiomes (Awasthi et al., 2020; Jo, Oh & Park, 2020). This technology can also perform gene function annotation (Awasthi et al., 2020; Jo, Oh & Park, 2020), thereby providing information on both the structural and functional characteristics of microbial communities among the different elevations. The Leigong Mountain mid-subtropical mountain forest ecosystem ranges from 650 to 2,179 m a.s.l., but our study focused on the 1,000 to 1,600 m a.s.l. range. Future studies on soil microbial community distribution patterns should disentangle the links between the environmental variables (vegetation types, edaphic factor, microclimate, *etc*.) and the detailed functions of the microbial communities along wider elevation gradients in this typical subtropical, non-karst mountain forest ecosystem.

Euryarchaeota and Thaumarchaeota were the predominant phyla of soil Archaea in the *P. massoniana* forest across the different elevations. Many reports have pointed out these phyla as the dominant in soil Archaea (Siles & Margesin, 2016; Wang et al., 2015), making important contributions to the biogeochemical cycles of soil carbon, nitrogen, and hydrogen, among many other elements. A good example of this paramount ecological function is that of autotrophic ammonia-oxidizing Archaea who perform a key step in the nitrogen digestion process in soils (Leininger et al., 2006; Zhang et al., 2012). The present study found that the abundance of Archaea had a lightly increasing trend with increasing altitude at 1,000–1,600 m a.s.l. on Leigong Mountain. This finding is consistent with results reported for the 1,000–1,500 m a.s.l. range on Mount Fuji, Japan (Singh, Takahashi & Adams, 2012).

Bacteria is the largest and most diverse and versatile of soil microbial groups (Delgado-Baquerizo et al., 2018). Consistent with the consensus that they are the most common phyla among soil bacteria (Wang et al., 2015), Proteobacteria and Acidobacteria were the predominant soil bacterial groups in the present study, both playing a very important role in the soil-matter cycle (carbon, nitrogen, sulphur, and other elements) and in the construction of the ecological environment (Barns et al., 2007; Spain, Krumholz & Elshahed, 2009).

Fungi are also an important component of the soil microbiome as they play a crucial role in nutrient cycling, as well as in promoting plant growth and vegetation community succession (Tedersoo et al., 2014). Consistent with previous reports (Matsuoka et al., 2019), the Ascomycota and Basidiomycota found in the present study were the predominant fungal groups across the altitudinal gradient under study; however, they showed opposite trends in relative abundance: the relative abundance of Ascomycota decreased with increasing elevation (from 66.9% at Mp1000 to 46.3% at Mp1600), whereas that of Basidiomycota increased with increasing elevation (from 25.2% at Mp1000 to 48.1% at Mp1600). Overall, the relative abundance of Ascomycota and Basidiomycota together increased slightly with elevation (92.2% at Mp1000 to 94.4% at Mp1600). The increasing trend in relative abundance observed herein for Basidiomycota is consistent with the increasing trend observed for the same group in at 950–1,700 m a.s.l. in Norikura Mountain, Japan (Ogwu et al., 2019). Furthermore, the trend observed here for Ascomycota was similar to that found in Norikura Mountain. Therefore, the specific set of predominant fungal groups may serve as a more sensitive indicator of environmental gradients determined by differences in altitude.

Viruses are ubiquitous and play important roles in regulating their hosts’ mortality and community structure, the genetic landscape, and the nutrient turnover in ecosystem (Anderson, Brazelton & Baross, 2011; Narr et al., 2017; Paez-Espino et al., 2016). Although soil is the most important habitat for virus distribution, there is currently a very limited number of reports on soil viruses (Narr et al., 2017; Paez-Espino et al., 2016), which may be due to the challenges involved in the detection, isolation, and classification of unknown viruses (Narr et al., 2017); therefore, viruses constitute what is referred to as “the dark matter” of soil microbial communities (Paez-Espino et al., 2016). In the present study, Myoviridae, Podoviridae, and Siphoviridae were the predominant virus groups in the soils of the *P. massoniana* forest of Leigong Mountain. Although they are reportedly the most common virus groups in a wide range of environments (Chen et al., 2014), we found no obvious trend in viral community composition changes between 1,000 and 1,600 m a.s.l. in the present study. This result likely reflects the fact that virus dynamics may be affected by multiple factors, including host and environmental factors. The results of the symbiotic network analysis further reflected the differences in interaction patterns among species within the different groups of the microbial community in a soil.

The gene functional composition of the soil microbial community at different altitudes was predicted by NOG, KEGG, and CAZy functional gene annotations. The results showed a relatively consistent trend among these different functional analyses. Thus, there were no obvious differences at the high category level in functional gene detection along the elevation gradient surveyed, a finding that may be related to the phenomenon of “gene redundancy” (Fierer et al., 2013; Louca et al., 2018). Functional redundancy of soil microbial communities may be a microbial community maintenance mechanism (ecological strategy), which can play an important buffer role in the process of combating external environmental changes and various disturbances (Louca et al., 2018). This emergent property of an open microbial system may be caused by the limitations of current research techniques (when a high-dimensional trait space is projected to a lower-dimensional function space of interest). Nonetheless, the comparison between the species composition matrix and the functional gene composition matrix of the soil microbial communities along the altitudinal gradient surveyed showed a significant correlation between the two. This supports our hypothesis that soil microbiome diversity can influence multiple ecosystem functions, while the elevation factor may indirectly influence soil microbiome functions by shaping soil microbiome composition.

Various factors, such as climate, vegetation type, plant diversity, and soil pH and other physicochemical properties, may determine the altitudinal distribution pattern of soil microbial diversity in a certain region (Bahram et al., 2018; Liu et al., 2018; Siles & Margesin, 2016). In this study, the investigated soil factors had a certain influence on the composition and function of soil microorganisms, but not significant (Fig. 8); the results may be due to the relatively small altitude span and the same forest vegetation. Considering there are still many limitations in the existing studies on the elevation gradient, such as the differences in the geographical location, size and habitat of the mountain, and the difference in the elevation span and the span size of vegetation types of the sampling points, the key drivers of the biological and abiotic environment of the soil microorganisms in the mountain ecosystem also are varied (Bahram et al., 2018; Liu et al., 2018; Siles & Margesin, 2016).

The co-occurrence networks of archaeal and fungal species showed a relatively high similarity, which may be related to the origin of the relationship between Archaea and fungi (Fig. 9). In turn, the co-occurrence network of bacterial and viral species showed a relatively high similarity, which may be due to the closer relationship between them in the life history of the individuals. Since complex soil processes are driven by soil microbiome including soil Archaea, bacteria, fungi, and viruses, these data indicate a challenging and complex ecological network of relationships among the different groups of soil microorganisms.

Overall, metagenomic sequencing technology in this study was used to comprehensively reflect structural and functional characteristics of soil microbial community in *P. massoniana* forest along different elevation gradients. However, due to the lack of survey site repetition, and not involving the time gradient (seasonal or inter-annual change) survey; Therefore, these aspects need to be further strengthened in future studies.

## Conclusions

Our findings suggest that different soil microbial communities (Archaea, Bacteria, Fungi, and Viruses) have different distribution patterns in a *P. massoniana* forest along an altitudinal gradient. This finding provide an ecological management and utilization guidance for incorporating soil microbial diversity and function in mid-subtropical mountain forest ecosystem.

## Additional Information and Declarations

### Data Availability

The following information was supplied regarding data availability: The sequencing data is available at NCBI SRA: PRJNA807058; SRR18029981, SRR18029982, SRR18029983, SRR18029984, SRR18029985.
